# Transformer- and Generative Adversarial Network–Based Inpatient Traditional Chinese Medicine Prescription Recommendation: Development Study

**DOI:** 10.2196/35239

**Published:** 2022-05-31

**Authors:** Hong Zhang, Jiajun Zhang, Wandong Ni, Youlin Jiang, Kunjing Liu, Daying Sun, Jing Li

**Affiliations:** 1 Guanganmen Hospital China Academy of Chinese Medical Sciences Beijing China; 2 School of Electronic Information Engineering Wuxi University Wuxi China; 3 Physician Qualification Program Certification Center of Traditional Chinese Medicine State Administration of Traditional Chinese Medicine Beijing China; 4 School of Electronic Engineering and Optoelectronic Technology Nanjing University of Science and Technology Nanjing China

**Keywords:** traditional Chinese medicine, transformer, generative adversary networks, electronic health records, artificial intelligence, natural language processing, machine learning, word2Vec

## Abstract

**Background:**

Traditional Chinese medicine (TCM) practitioners usually follow a 4-step evaluation process during patient diagnosis: observation, auscultation, olfaction, inquiry, pulse feeling, and palpation. The information gathered in this process, along with laboratory test results and other measurements such as vital signs, is recorded in the patient’s electronic health record (EHR). In fact, all the information needed to make a treatment plan is contained in the EHR; however, only a seasoned TCM physician could use this information well to make a good treatment plan as the reasoning process is very complicated, and it takes years of practice for a medical graduate to master the reasoning skill. In this digital medicine era, with a deluge of medical data, ever-increasing computing power, and more advanced artificial neural network models, it is not only desirable but also readily possible for a computerized system to mimic the decision-making process of a TCM physician.

**Objective:**

This study aims to develop an assistive tool that can predict prescriptions for inpatients in a hospital based on patients’ clinical EHRs.

**Methods:**

Clinical health records containing medical histories, as well as current symptoms and diagnosis information, were used to train a transformer-based neural network model using the corresponding physician’s prescriptions as the target. This was accomplished by extracting relevant information, such as the patient’s current illness, medicines taken, nursing care given, vital signs, examinations, and laboratory results from the patient’s EHRs. The obtained information was then sorted chronologically to produce a sequence of data for the patient. These time sequence data were then used as input to a modified transformer network, which was chosen as a prescription prediction model. The output of the model was the prescription for the patient. The ultimate goal is for this tool to generate a prescription that matches what an expert TCM physician would prescribe. To alleviate the issue of overfitting, a generative adversarial network was used to augment the training sample data set by generating noise-added samples from the original training samples.

**Results:**

In total, 21,295 copies of inpatient electronic medical records from Guang’anmen Hospital were used in this study. These records were generated between January 2017 and December 2018, covering 6352 types of medicines. These medicines were sorted into 819 types of first-category medicines based on their class relationships. As shown by the test results, the performance of a fully trained transformer model can have an average precision rate of 80.58% and an average recall rate of 68.49%.

**Conclusions:**

As shown by the preliminary test results, the transformer-based TCM prescription recommendation model outperformed the existing conventional methods. The extra training samples generated by the generative adversarial network help to overcome the overfitting issue, leading to further improved recall and precision rates.

## Introduction

The widespread use of electronic health record (EHR) systems has led to the explosive growth of digitized health care data. As the amount and complexity of data grow, medical analysis and decision-making become increasingly time-consuming and error prone. In reality, a human physician cannot fully use all the available information at his or her disposal in a timely fashion. Therefore, harnessing the information contained in EHR data, most of which is in textual form, is critical for driving innovation research, improving health care quality, and reducing costs. Natural language processing (NLP) is essential for transforming relevant information sequestered in freestyle texts into structured data for further computerized processing. The development of a predictive model with EHR data was motivated by the desire to offer a medication-oriented decision support tool to clinical health care providers. To build such a predictive model, we used NLP techniques to convert a patient’s EHR data into a representation, which then becomes the input to a deep learning model to predict medical events, such as medication orders.

Biomedical NLP has experienced great progress in the past 30 years [1,2] and has become especially active in recent years [3]. Previously, EHR data were analyzed using traditional machine learning and statistical techniques such as logistic regression, support vector machine, and random forest [4]. However, in recent years, as reviewed in the studies by Shickel et al [5], Sheikhalishahi et al [6], and Miotto et al [7], many research efforts have been devoted to the application of deep learning techniques to EHR data for clinical informatics tasks. Autoencoders have been used by researchers [8] to predict a specific set of diagnoses. A long short-term memory (LSTM) sequence model [9] was trained to provide patient-specific and time-specific predictions of medication orders for patients who are hospitalized [10]. A convolutional neural network (CNN) model was used to predict discharge medications using the information available at admission [11]. Numerous articles were surveyed in the study by Goldstein et al [12] regarding the development of a risk prediction model using EHR data. A comprehensive study on applying deep learning techniques to EHR data for a variety of prediction problems was reported in the study by Rajkomar et al [13]. Recurrent neural networks were successfully trained using EHR data to detect medical events [14-16].

The research on applying artificial intelligence in traditional Chinese medicine (TCM) has been very active in the past decade [17,18]. Data mining techniques have been used for TCM syndrome modeling and prescription recommendation for diabetes [19]. The PageRank algorithm [20] was modified and applied to TCM prescription recommendations [21]. In our previous work [17], a CNN was used to predict TCM diseases, and XGBoost, along with other neural networks, was used to predict TCM syndromes. Following the sequence-to-sequence paradigm, researchers from Peking University used bidirectional gated recurrent neural networks to generate TCM prescriptions from symptom descriptions [22]. They proposed a coverage mechanism along with a soft loss function as a remedy for the repetition problem they encountered. However, the requirement of curated descriptions of symptoms as inputs hinders the practicality of this approach. Ideally, the model generates TCM prescriptions directly from raw EHR data, similar to how a human TCM physician conducts deductive reasoning.

Generating prescriptions from raw EHR data typically comprises 2 parts. The first part uses biomedical NLP [3] techniques to extract relevant information used by a human physician to form a feature representation [23]. The second part uses deep learning techniques [7] to map this feature representation into a prescription order.

The primary task of biomedical NLP is to extract relevant information from clinical narratives written in free-form text and store the gathered information as structured data. Numerous deep learning techniques [24-26], such as bidirectional LSTM (BiLSTM), have been used in the biomedical NLP field. Both BiLSTM conditional random field (CRF) and transformer CRF have been used for named entity recognition (NER) of EHR notes written in Chinese [27,28]. The recognized entities are then formed into distinct tokens. Then, the feature representation of a patient’s EHR data becomes a sequence of tokens. The tokens are then converted into real-valued multidimensional vectors using word embedding techniques [29].

The purpose of this study was to develop an assistive tool that can prescribe TCM prescriptions for inpatients in a hospital based on the patient’s clinical EHRs. The predictive model for TCM prescription generation is based on a sequence-transducing model called the transformer [30]. This model is entirely based on attention, replacing the recurrent layers most commonly used in encoder-decoder architectures with multihead self-attention. The training used in this predictive model was supervised training with human-authored prescriptions contained in the EHR data set as the training targets. Furthermore, a generative adversarial network (GAN) [31] model was designed to augment the training set to further enhance the overall system performance by reducing the effects of overfitting.

## Methods

This section is arranged as follows: the overall system architecture is briefly described; then, each constituent subsystem, which may comprise some functional blocks, is introduced; finally, the training process is described in the *Training* subsection, where a GAN model was used to generate noise-added samples from the original samples.

### System Overview

Hospitals and medical institutes in China are rapidly moving toward standardizing their EHRs to conform to the regulations and specifications issued by the Ministry of Health of the People’s Republic of China [32-34]. A standard EHR document for a patient may contain up to 53 parts, depending on the patient’s situation. These may include the following:

- A first page record containing the patient’s basic personal information, such as sex, age, occupation, and marital status
- An admission record containing the description of a patient’s illness upon admission to the hospital, including chief complaints, medical history, and family medical history
- A laboratory tests record containing the list of tests and the corresponding results
- A nursing record containing nurse notes of the patient’s condition, treatments taken and nursing care taken, body temperatures and vital signs taken, and physician’s orders
- A treatment procedure record containing the entire in-hospital diagnosis and treatment process and any changes to the patient’s illness or illnesses

**Figure 1:**
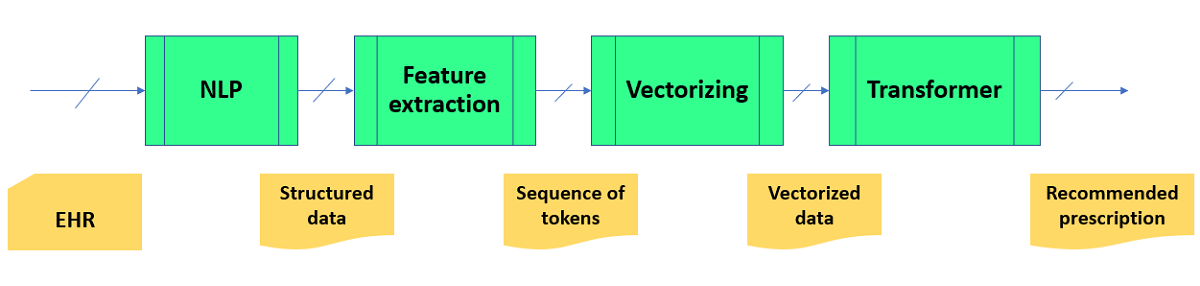
Block diagram of the prescription generation system. EHR: electronic health record; NLP: natural language processing.

A high-level block diagram of the proposed system is shown in Figure 1. The system comprises 4 subsystems: the NLP subsystem, the feature extraction subsystem, the vectorization subsystem, and the prescription prediction subsystem. The NLP subsystem processes the EHR file and produces structured data, which in turn are processed by the feature extraction subsystem to extract relevant clinical information for prescription prediction. The vectorization subsystem maps the sequence of tokens written in Chinese characters to digital numbers, presented as a vector in a multidimensional space. The prescription prediction subsystem, which is a transformer-based deep learning model, automatically generates a prescription based on input vector data. Together, the first 3 subsystems accomplish the task of extracting relevant information from an EHR file to form input variables for the prediction model. Similar representation learning operations were described in our previous paper [17].

In short, NLP normalizes the raw EHR data, the feature extractor converts the normalized data into a sequence of tokens, the vectorization subsystem maps the tokens into vectors of real numbers, and the predictive model performs the reasoning process to produce a prescription.

### The NLP Subsystem

**Figure 2:**
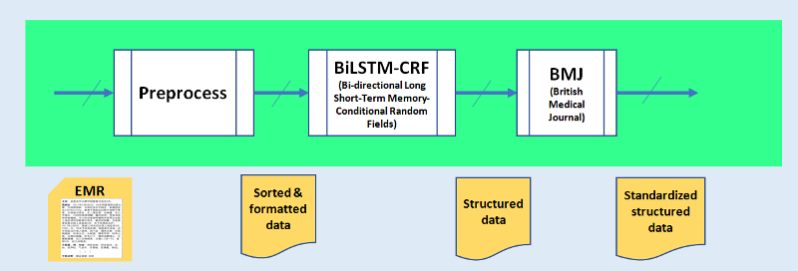
Block diagram of the named entity recognition subsystem. BiLSTM: bidirectional long short-term memory; EMR: electronic medical record; BiLSTM-CRF: Bidirectional long short term memory – conditional random fields; BMJ: British Medical Journal.

This subsystem is responsible for generating structured data from original EHR documents. The internal block diagram of the subsystem is shown in Figure 2. There are 3 functional blocks in this subsystem: the preprocessing block, NER block, and British Medical Journal block.

The preprocessing block cleans the raw EHR document by removing pictures and unusable components. This ensures the completeness and accuracy of the electronic medical records. Electronic medical records with incomplete or inconsistent information are discarded.

After the initial cleaning, the content of the EHR file is then divided into distinct sections. For example, the admission record is divided into sections of chief complaints, medical history, and others. Then, all the resultant sections are sorted, formatted, and subsequently fed to the NER block.

Only a small part of the EHR document is in a fixed format, and the remainder is in unstructured freestyle narratives. For fixed-format texts, a script is used to extract named entities to form structured data.

For freestyle narratives, a functional block called entity recognition is used to extract named entities to form structured data entries. The NER block is implemented using a BiLSTM network with CRF (BiLSTM-CRF) [24].

Then, the extracted named entities such as symptoms, illness, medicine, examinations, and tests are further standardized according to a Chinese version of the British Medical Journal Best Practice knowledge base.

**Figure 3:**
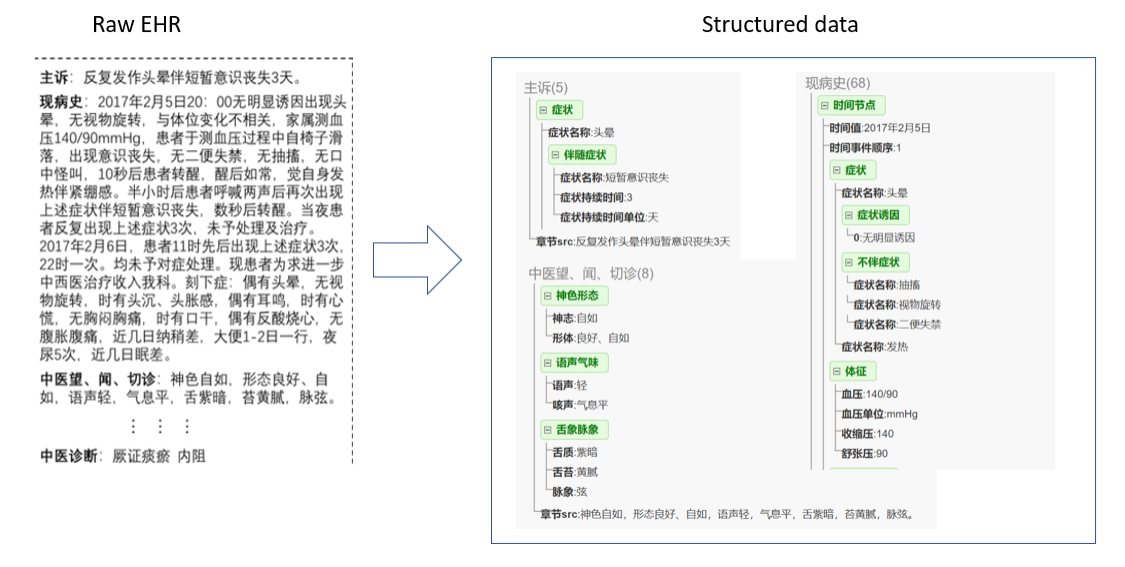
Example of converting a freestyle narrative into structured data. EHR: electronic health record.

Figure 3 shows an example of the processing result, where the admission record of a raw EHR note is converted into structured data, with the marked words being named entities.

### The Feature Extraction Subsystem

To effectively mimic the reasoning process conducted by a human physician, accurate and relevant input variables must be chosen properly. These variables should represent the complete set of factors that a human physician should take into consideration when making treatment decisions. Textbox 1 summarizes the predominant factors that TCM experts consider when making treatment decisions.

**Figure 4:**
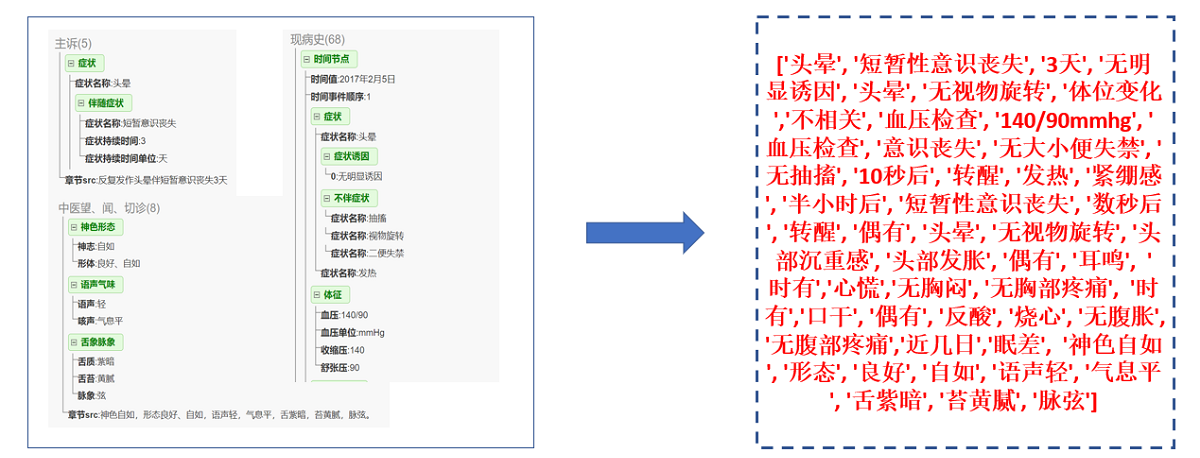
Example of converting structured data into a sequence of tokens.

The feature extraction subsystem extracts the aforementioned key features from the standardized structured data to form a sequence of tokens. Figure 4 shows an example of this feature extraction, in which a sequence of tokens is generated from structured data.

**Text type and the content to extract.**

**Demography**

- Sex, age, height, weight, and BMI

**Chief complaints**

- Symptoms and signs

**Recent medical history**

- Symptoms, signs, and general information

**Past medical history**

- Past illness and medicines taken

**Present illness**

- Tongue coating and pulses

**Body check**

- Vital signs

**Treatment process records**

- Current illness situation and treatment plan

**Physician’s orders**

- Prescriptions

**Nursing notes**

- Vital signs and medication records

**Examination reports**

- Examination items and findings

**Laboratory reports**

- Items tested and qualitative and quantitative test results

### The Vectorization Subsystem

#### Overview

Until this point, all medical information needed to make a treatment decision was encapsulated in textual data expressed in Chinese characters. To be used by the deep learning network—the Transformer—the information must be mapped into a digital variable. In this vectorization process, a Chinese word or phrase is represented as a real-valued vector in multidimensional feature space. This section explains how tokenized features are further processed through word embedding.

#### Training the Word Embedding Model

The corpus was a collection of 102,596 electronic medical records from Guang’anmen Hospital and other hospitals. The *Jieba* tokenizer was used to perform tokenization. The open-source modeling tool *Gensim* was used to train the word2vec [29] model with the following major parameters: *min_count*=2, *vector_size*=100, *window*=5, *sg*=1, *hs*=1, and *epochs*=50.

The Skip-Gram model was used, as indicated by the parameters. Each word was represented by a real-valued vector of 100 dimensions.

#### Vectorization

Once the word embedding model is trained, each token is represented by a 100-dimension vector. For each word in the input sequence, a unique identifier is assigned using a numerical-type value expressed as a name-value-unit before another unique identifier is assigned. Once all tokens are converted into vectors, the vectors are then concatenated to form a single vector variable, which then serves as the input to the transformer.

**Figure 5:**
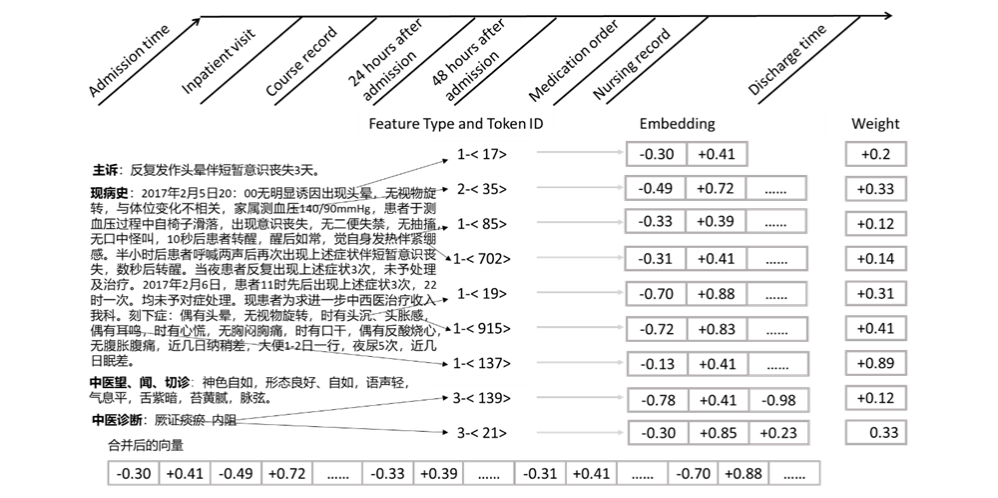
Illustration of converting electronic health record text to word vectors.

The NLP, feature extraction, and vectorization subsystems together accomplish the task of feature learning by converting an EHR document into a multidimensional real-valued vector. Figure 5 shows an example of mapping from EHR text to word vectors.

### The Transformer Subsystem

**Figure 6:**
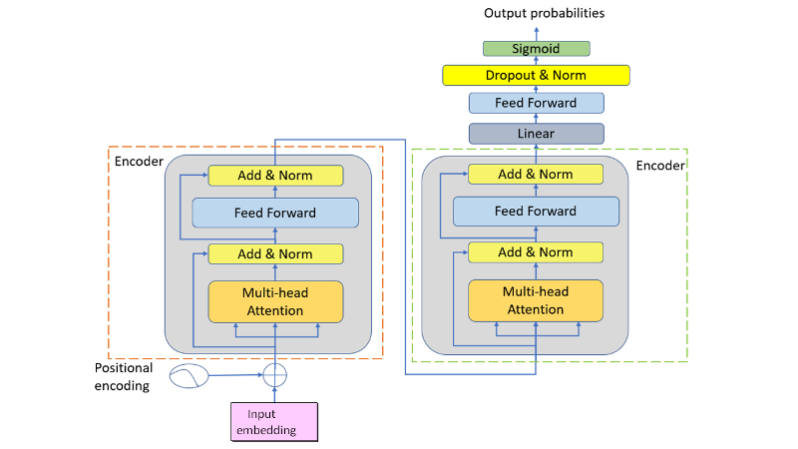
The transformer subsystem.

The transformer subsystem is responsible for recommending a prescription for every given input embedding, as shown in Figure 6. The subsystem is described in the following paragraphs.

Input embedding is a vector of *max_num_tokens× vector_size* dimensions. For example, *max_num_tokens*=759 and *vector_size*=100*.* Zero padding is used if the number of tokens in a sequence is smaller than *max_num_tokens.* Conversely, if the number of tokens in a sequence is larger than *max_num_tokens*, the number of tokens is capped at *max_num_tokens* by dropping off tokens corresponding to the oldest time stamp with respect to the current prescription generation time. The input embedding sample is first added to the position vector of the same size, becoming the input to the first encoder.

The main body of the subsystem comprises 2 identical cascaded transformer encoders. Unlike the encoder of the original transformer [30], which comprises 6 identical layers, the encoder used in this research had only 1 layer with 4 sublayers. The first was a multihead self-attention layer with *Multi_heads*=4 and *head_dim*=8. The second was a residual layer of 100 neurons with normalization. The third was a simple, position-wise, fully connected feedforward network of 2048 neurons. The fourth was a residual layer of 100 neurons with normalization.

The second encoder was followed by a linear layer, a feedforward layer of 2048 neurons, a hidden layer, and an output layer, as shown in Figure 6. The output layer comprised 819 neurons with a sigmoid activation function. Each of the 819 neurons corresponded to an herbal ingredient. The hidden layer comprised 128 neurons with a dropout mechanism and normalization. The dropout rate was set to 0.4740. The purpose of this hidden layer was to prevent overfitting.

The final result from the output layer was a list of probabilities for the 819 drug ingredients, valued between 0 and 1. The recommended prescription was then obtained by setting a threshold for these probabilities.

### Training

#### Training the Transformer

Training of the transformer is a supervised learning process. The input is a real-valued vector representation of a patient’s EHR, and the output is the prescription. The learning goal is for a machine-generated prescription to match the medical order prescribed by a human physician.

#### Augmenting the Training Data

To alleviate the overfitting effect of the proposed prediction model, a GAN [31] network was used to augment the training data set. Following the fundamental idea of the GAN network, the generative model G is trained to represent the distribution of the original training data set, and the discriminative model D is trained to detect whether the sample originates from the original sample set or from the output of the generative model.

**Figure 7:**
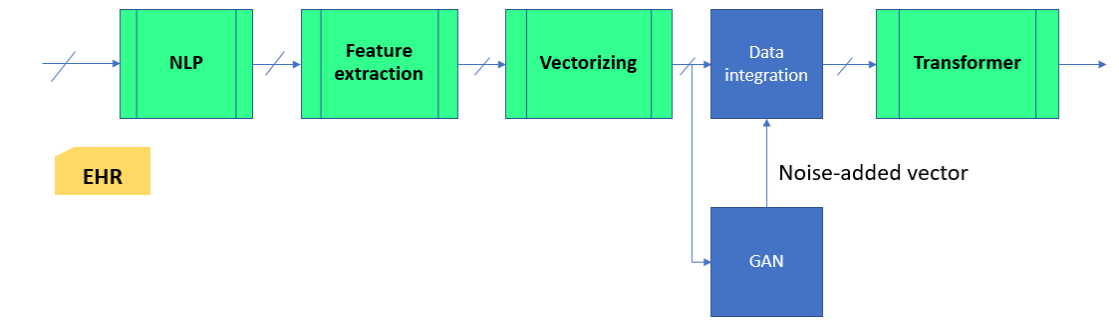
Block diagram of the predictive modeling system during the training phase. EHR: electronic health record; GAN: generative adversarial network; NLP: natural language processing.

During the training phase, the entire system looks like that shown in Figure 7. For every original training sample, there is a noise-added sample. The use of a GAN in this system effectively doubled the number of training samples.

**Figure 8:**
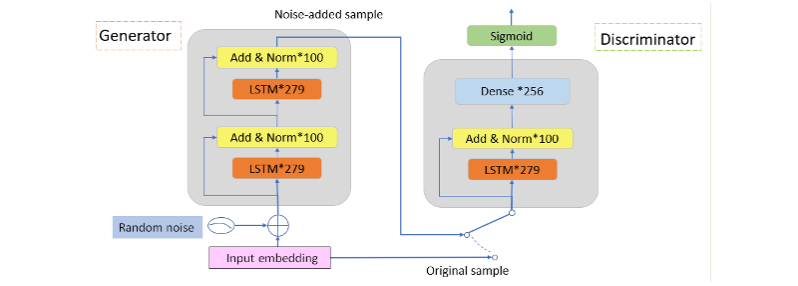
The internal structure of the generative adversarial network subsystem. LSTM: long short-term memory; *size of the neural network used in that layer.

The internal structure of our GAN network was designed as shown in Figure 8. Generator G comprises 2 identical LSTM layers, each with a size of 279. Each LSTM layer is followed by a normalization layer with a residual connection. The input to the discriminator G could be either an original word embedding sample or a noise-added sample generated by the generator G. The discriminator D comprises an LSTM layer with a size of 279, a residual and normalization layer with a size of 100, and a full connection layer with a size of 256. Finally, the discriminator D outputs a binary value using a sigmoid function.

We followed a typical GAN network training procedure [31] to train the GAN subsystem, simultaneously training the discriminator and generator. The discriminator and generator alternate in their training until a Nash equilibrium is reached.

The generator first produces a *batch_size* noise-added EHR, embedding samples with randomly initialized coefficients of the generator network. These samples are concatenated with the original noise-free EHR embedding samples to form (2×*batch_size*) embedding samples, each with *max_num_tokens×vector_size* real values. For example, we can have *batch_size*=500, *max_num_tokens*=560, *vector_size*=100. These (2×*batch_size*) samples were used as inputs to the discriminator. For every input sample, an output label indicates whether the sample is from the true original embedding or from the generator. The discriminator network was trained using a backpropagation algorithm with the objective of minimizing the prediction error. The training of the discriminator is halted when the binary cross-entropy loss function stops decreasing. The discriminator training is then temporarily halted to yield to the generator training.

To train the generator, all network coefficients of the discriminator must be frozen. The discriminator now works in tandem with the generator during generator training. The generator produces *batch_size* noise-added embedding samples, and for every sample, the discriminator outputs a prediction. The generator updates its parameters using a backpropagation algorithm based on the discriminator output. The training of the generator is halted when the binary cross-entropy loss function stops increasing. The generator training is then temporarily halted to yield the discriminator training.

The aforementioned discriminator and generator training processes together form 1 training epoch. The entire GAN network training is accomplished through several epochs. The training stops when a Nash equilibrium is reached.

The entire training process is illustrated using the Python pseudocode included in Multimedia Appendix 1.

### Ethics Approval

This study received institutional review board review through Guanganmen Hospital Ethic Committee (SQ2017YFGX 060073).

## Results

### Data Set

EHRs generated in Guang’anmen Hospital between January 1, 2017, and December 31, 2018, were used as the data set in this study. Initially, there were 27,846 copies of EHR notes, out of which 6551 (23.53%) copies were discarded because of quality control. An EHR note should be discarded if it satisfies one of the following conditions:

- The note is incomplete for missing certain basic pages.
- The note contains inconsistent information.
- The note does not use standard descriptions.
- The note contains special EHR circumstances such as chemotherapy, after an operation, and removal of fracture settings.

### Evaluation Metrics

The data set contained 6352 drug varieties. A complete TCM prescription includes drug ingredients, dosages, and decoction preparation instructions. It is still very challenging, if not impossible, for a machine to generate such a complete TCM prescription. At our current stage of research, we focus only on the drug ingredients of a prescription.

**Figure 9:**
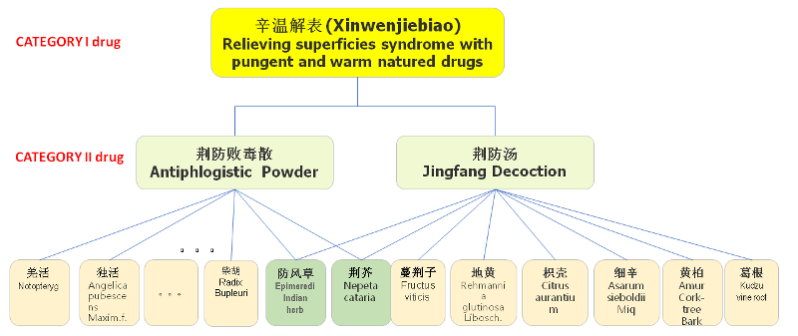
Classification of herbal drugs.

Judging whether the 2 TCM prescriptions are the same is often not straightforward, given the distinctive nature of TCM [35]. Often, 2 different herbs may have the same medical effect. When a TCM physician prescribes a medication order, he or she often has multiple choices at hand for herbal ingredients. As a result, the 2 TCM physicians may prescribe different herbs for the same patient with the same diagnosed condition. Therefore, it is necessary to have a unified method of evaluating machine-generated prescriptions. To this end, we need a higher level of abstraction. Figure 9 shows an example of the organization of TCM drugs. In this example, 2 TCM drugs (antiphlogistic powder and Jingfang decoction) have different herbal ingredients but belong to the same parent drug category and have the same medical treatment effect. In our research, we concluded that the recommended drug should be considered a correct recommendation as long as the recommended drug belongs to the same parent category as that of the human-authored prescription.

To quantitatively evaluate the performance of the transformer-based deep learning model, we compared the prescription generated by the machine with that prescribed by a human physician. Here, we used the metrics of *precision rate* and *recall rate*, which we based on 3 variables. True positive (TP) is defined as the number of drugs that exist in the physician’s prescription and also exist in the machine’s prescription. False positive (FP) is the number of drugs that do not exist in the physician’s prescription but exist in the machine’s prescription. False negative (FN) is defined as the number of drugs that exist in the physician’s prescription but not in the machine’s prescription. With these definitions, we defined the precision and recall rates as follows:

Precision rate = TP / (TP + FP) **(1)**

Recall rate = TP / (TP + FN) **(2)**

### Hyperparameter Tuning With GridSearchCV

The data set was divided into training and test sets, with the training set comprising 90% of the data set and the test set comprising the rest. The model was trained using a 10-fold cross-validation method; that is, the training set was randomly split into 10 folds, with the model being trained 10 times. During each of the 10 training times, the hyperparameters were tuned using the GridSearchCV method. Each training resulted in a set of hyperparameters, with the ultimate hyperparameters being the average of these 10 sets of parameters.

The values of the hyperparameters of the transformer network model have a great influence on the accuracy of the model. The optimal values of these parameters were determined through iterations using the grid search method. The sparse characters of each type were embedded into a d-dimensional embedding layer. Then, all vectors were combined using a new method: vectors of the same type and time were averaged using the weights of self-learning.

The model was optimized using a minimal log loss. Many regularization methods were used, such as the vector loss rate and the embedded layer loss rate. In addition, small-scale L2 weight punishment was used, which increased the punishment for large weights. The training batch size was chosen as 128, placing sentences with similar sizes into the same batch. Each batch contained approximately 12,000 words. Finally, the multilabel task was processed using an Adam function. For multilabel tasks, the input with the last time stamp was multiplied with the special end of sequence embedding. The training was executed using the Kears framework on a server with 8 NVIDIA P100 graphics processing unit. The fine-tuned hyperparameters along with their respective ranges are shown in Table 1.

**Table 1:** Some hyperparameters of the model.

Hyperparameters	Values	Parameter range
Gradient	0.1245	(0.1, 0.5, 1.0, 1.1)
Attention heads	4	(4, 8)
Vector loss rate	0.4410	(0.25, 0.35, 0.5)
Hidden layer loss rate	0.4740	(0.25, 0.35, 0.5)
Learning rate	0.4375	(0, 1)
L2 punishment rate	0.000001566	(0, 0.01)

### Experimental Results

**Figure 10:**
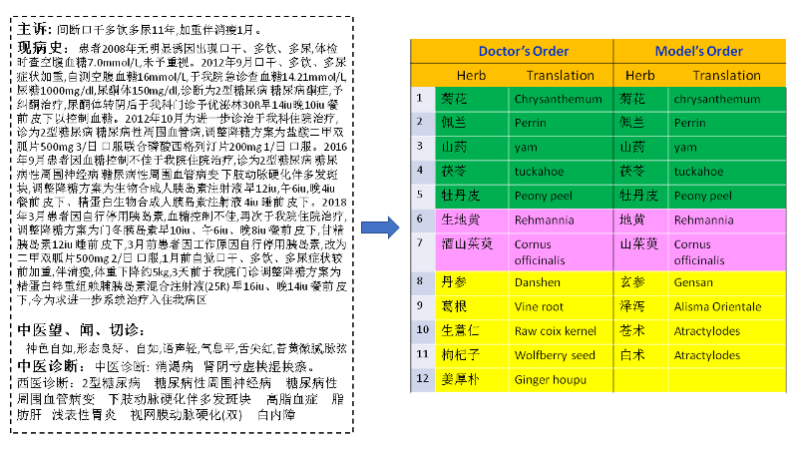
Side-by-side comparison of physician’s order versus model’s order.

To intuitively explain our experimental results, we start with a concrete example that illustrates how EHR notes lead to prescription orders. An example of this is shown in Figure 10. The left side shows a snapshot of the patient’s EHR. On the right side is a table showing a side-by-side comparison between a human-authored order and the prescription generated by our model. The physician’s order contains 12 ingredients, whereas the model’s order has 11. The first 5 ingredients are identical on both sides. The sixth ingredient from each side is the same, although they have different Chinese names. This is because the physician used a nickname for the herb. The remaining ingredients differ not only in name but also in substance. However, these 2 orders are still considered equivalent so far as the medical treatment effect is concerned. This is because in TCM terminology, a diagnosis must conclude with the name of the disease (illness) and a list of syndromes [17]. In this particular case, the diagnosed disease is *emaciation-thirst*, with the primary syndrome being *kidney and liver deficiency* and the secondary syndrome being *dampness and stasis*. The first 6 herbal ingredients target the primary syndrome. The remaining ingredients in each prescription are for the treatment of the secondary syndrome called *dampness and stasis*. As these 2 orders are only slightly different in their ingredients for treating secondary syndrome, they are treated as the same prescription in our research.

**Figure 11:**
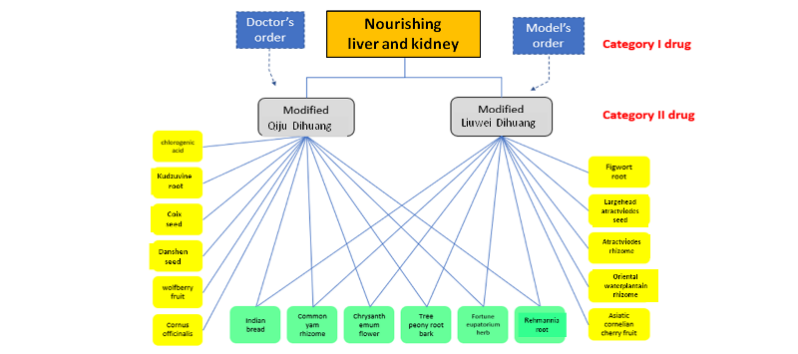
Prescription comparison: physician’s order versus model’s order.

To further explain this prescription comparison, we present another picture, as shown in Figure 11. The physician’s order is called *Qiju Dihuang pill*, and the model’s order is called *Liuwei Dihuang pill*. They are category II prescriptions that belong to the same parent category TCM prescription called *nourishing liver and kidney*. They differ only in how to dispel dampness and resolve phlegm to address only the secondary syndrome.

To evaluate the performance of the transformer-based predictive model, we first conducted model training using only the original samples, purposefully excluding the noise-added samples. The results are described in the following paragraphs.

On the basis of the time sequences, the system produced prescription recommendations at admission, 24 hours after admission, 48 hours after admission, 3 days after admission, and 1 week after admission. The test results are shown in Table 2.

From Table 2, we first observe that the precision and recall rates obtained from the training data set are higher than their respective counterparts from the test data set. This is understandable as the model has seen the samples from the training data set before but not from the test data set. The second observation is that as time progresses, both the precision and recall rates improve. After admission, at each subsequent medication order time, more relevant information is collected, and the prediction becomes more accurate. Although the number of feature tokens was <260 for 98% of the patients at the time of admission, this number increased to 296 in 24 hours, 333 in 48 hours, 366 in 72 hours, and 759 in 7 days. In our experiment, we set *max_num_tokens*=759*.* This means that when the number of feature tokens was <759, zero padding was used, and clipping was used when there were >759 feature tokens. Selecting the proper value for *max_num_tokens* is important for balancing the trade-off between overall system performance and computational efficiency. If the value is too large, training and inferencing will consume too much computation horsepower. If the value is too small, then some critical information gathered at admission will be lost because of clipping, leading to reduced precision and recall rates for prescription predictions at a time that is far from the admission time (eg, 2 weeks after admission).

The second set of experimental results was obtained using more training samples to train the predictive model. The size of the training data set was doubled, as for every training sample, a noise-added sample was generated by the GAN network. The precision and recall rates are listed in Table 3.

As can be seen in Table 3, both the precision and recall rates consistently improved by a noticeable margin. The results convincingly prove that inserting noise-added training samples generated by the GAN module can effectively overcome the overfitting issue, leading to better prediction performance.

**Table 2:** The precision rates and recall rates with transformer only.

Time	Training set		Test set	
	Precision rate (%)	Recall rate (%)	Precision rate (%)	Precision rate (%)
Admission	81.58	69.49	73.82	61.25
In 24 hours	83.37	71.88	74.56	62.69
In 48 hours	83.92	71.26	74.81	63.04
In 3 days	85.16	73.89	76.24	65.38
In 1 week	87.02	75.17	77.94	67.15

**Table 3:** The precision rates and recall rates with transformer+generative adversarial network.

Time	Training set		Test set	
	Precision rate (%)	Recall rate (%)	Precision rate (%)	Recall rate (%)
Admission	82.22	70.65	80.58	68.49
In 24 hours	84.15	72.18	82.37	70.8
In 48 hours	84.32	72.56	82.92	70.26
In 3 days	87.04	75.10	85.04	74.38
In 1 week	88.91	76.79	86.82	76.23

### Comparison Study

To compare the performance of our proposed model with that of existing prescription generation models, we implemented 3 other models. The CNN-based model [11] comprises a word embedding layer, a convolution layer that contains 3 filters of different sizes, a pooling layer, and a full connection layer. The output layer contains 819 neurons, equal to the number of prescribed herb varieties. The seq2seq [36] model comprises a CNN encoder and an LSTM decoder. The MedAR [37] model comprises a word embedding layer, followed by an attention layer, and finally, a RethinkNet layer to complete the multilabel classification. The learning rate was 0.001, the dropout rate was 0.8, and the optimization function was Adam. The final output layer used the sigmoid function, where all other layers used the non-linear activation function ReLU, which outputs an input x as zero if x is negative, and outputs x itself if x is larger than or equal to zero. Table 4 shows the respective precision and recall rates at admission for all 4 models in discussion. The results suggest that the proposed model has superior performance in terms of precision and recall rates.

**Table 4:** Performance comparison for different models.

Model	Precision rate (%)	Recall rate (%)
Convolutional neural network	47.54	31.00
Seq2seq^a^	64.02	48.74
MedAR^b^	71.46	53.08
Transformer+generative adversarial network	80.58	68.49

^a^Seq2seq: sequence to sequence model.

^b^MedAR: Medical data attention Rethink Net.

## Discussion

### Principal Findings

The following tasks have been finished in this research:

1. Deep learning NLP techniques were used to convert raw Chinese EHR texts into feature representations.
2. The major contribution of this study is the proposal of a transformer-based predictive modeling scheme for medication order generation from a feature representation of EHR data.
3. The secondary contribution of this study is the use of GAN to augment the training data set, leading to a noticeable performance improvement of the predictive model. Using the GAN, noise-added samples were generated to double the number of original training samples. This helped alleviate the overfitting problem, making the model more robust in terms of generalization.

### Limitations

Despite the efforts made in many aspects of the diagnosis and treatment scheme recommendations, there is still much room for improvement. The training data set is still relatively small, and there may be some frequently used medicines that are not included in the training data set. The TCM prescription knowledge base is still incomplete. Some medicines do not have standard names, and no corresponding parent medicine name exists in the database. Therefore, the recommended medicine names are still the original hospital medicine names. For a multilabel prediction task, an increased number of labels will increase the difficulty of the model prediction and lower the prediction accuracy. Therefore, as a more complete knowledge base is developed, the label set will be further optimized, leading to a greater prediction accuracy of the model.

### Future Work

This paper reports the preliminary research results of automated medication order generation from EHR texts for TCM inpatients who are hospitalized. The recommended medicines include Western and Chinese medicines. For Chinese medicines, only the medicine names are recommended. In the future, the dosage of the herbal ingredients, as well as the medicine preparation instructions, will be included in the recommendations. Improving the model prediction accuracy to the level of category II is also a direction for future work. Future work could expand the training data set to optimize the model.

## Appendix

Multimedia Appendix 1 Python pseudocode for the training of generative adversarial network with Keras Framework.

## Abbreviations

BiLSTM: bidirectional long short-term memory
CNN: convolutional neural network
CRF: conditional random field
EHR: electronic health record
GAN: generative adversarial network
LSTM: long short-term memory
NER: named entity recognition
NLP: natural language processing
TCM: traditional Chinese medicine
