# Supplementary material for: Transformer- and Generative Adversarial Network–Based Inpatient Traditional Chinese Medicine Prescription Recommendation: Development Study
Source: JMIR Med Inform. 2022 May 31;10(5):e35239. doi: 10.2196/35239 (PMC9198826; doi:10.2196/35239)
Supplement: Multimedia Appendix 1 [file medinform_v10i5e35239_app1.docx]

# appendix

**Python pseudo code for the training of GAN network with Keras framework**

*batch_size* = 500

# generator model

d = self.discriminator_model()

# discriminator model

g = self.generator_model()

# generator with discriminator model

d_on_g = self.generator_containing_discriminator(g, d)

# optimization function for generator with a discriminator

d_on_g.compile (loss='binary_crossentropy', optimizer=SGD(lr=0.0005, momentum=0.9, nesterov=True))

# optimization function and discriminator loss function

d.compile(loss='binary_crossentropy', optimizer= SGD(lr=0.0005, momentum=0.9, nesterov=True))

for *epoch* in *EPOCH_RANGE*

# training discriminator

d.trainable = True

*real_EHR_batch* = self.x_sample (*batch_size* = *batch_size*)

*noise_EHR_batch* = self.z_sample (*batch_size* = *batch_size*)

*generated_EHR_batch* = g.predict(*Real_EHR_batch*，*noise_EHR_batch*, verbose=0)

*input_samples_x* = np.concatenate((*real_EHR_batch*, *generated_EHR_batch*))

*output_labels_y* = [1] * batch_size + [0] * batch_size

d_loss = d.train_on_batch(*input_samples_x*, *output_labels_y*)

*noise* = self.z_sample(batch_size=batch_size)

# training generator

d.trainable = False

*fake_y* = [1] * *batch_size*

g_loss = d_on_g.train_on_batch(*real_EHR_batch*，*noise*, *fake_y*)

end for
